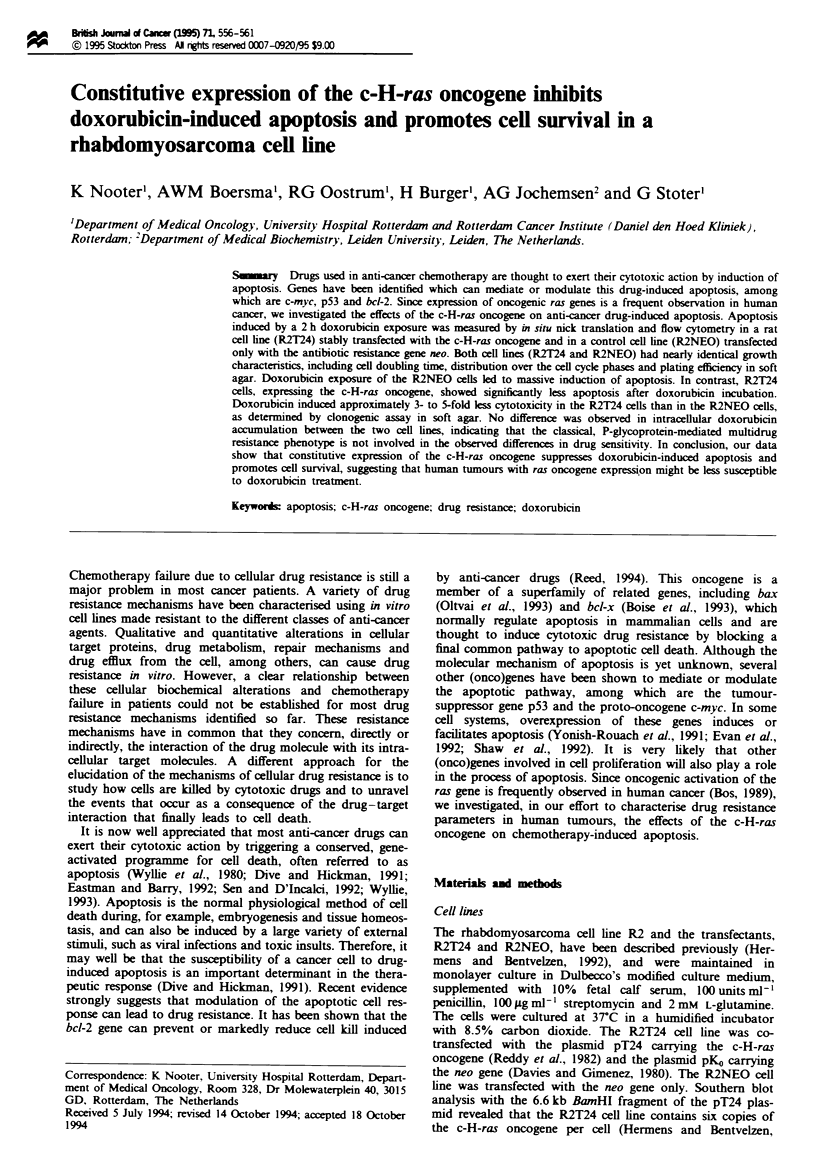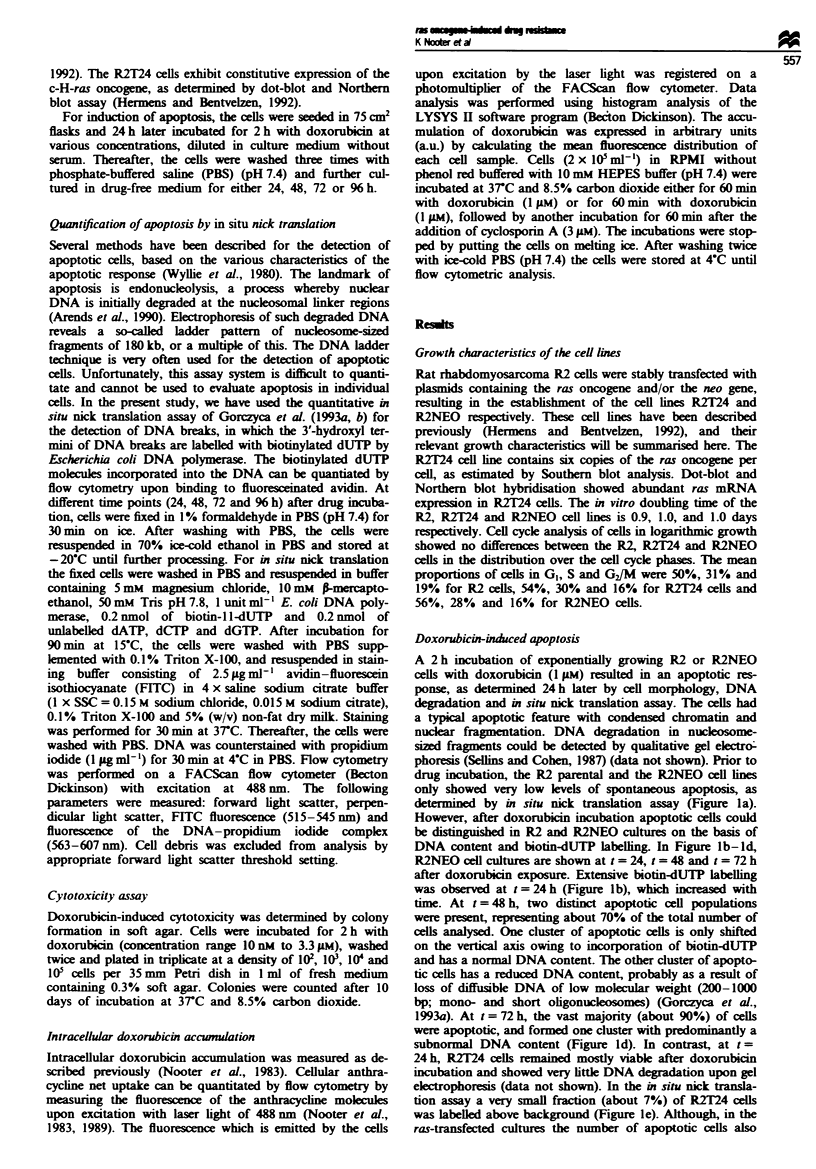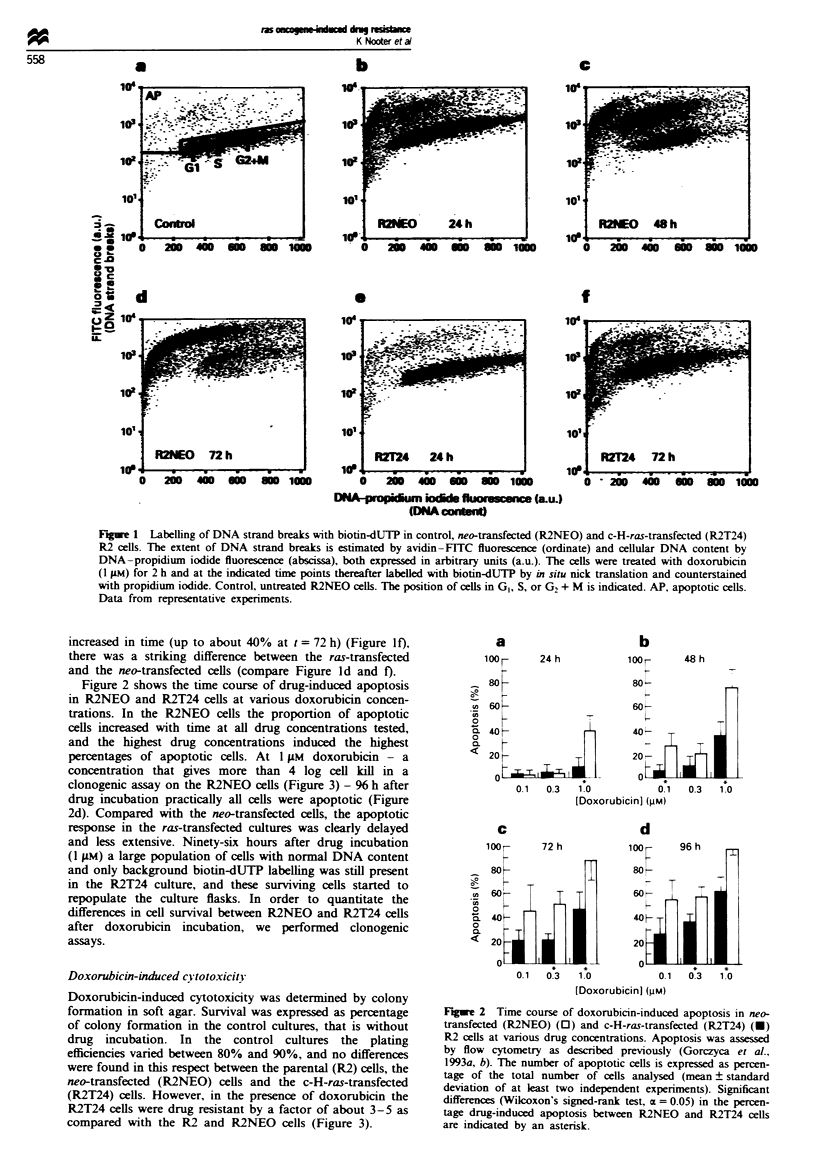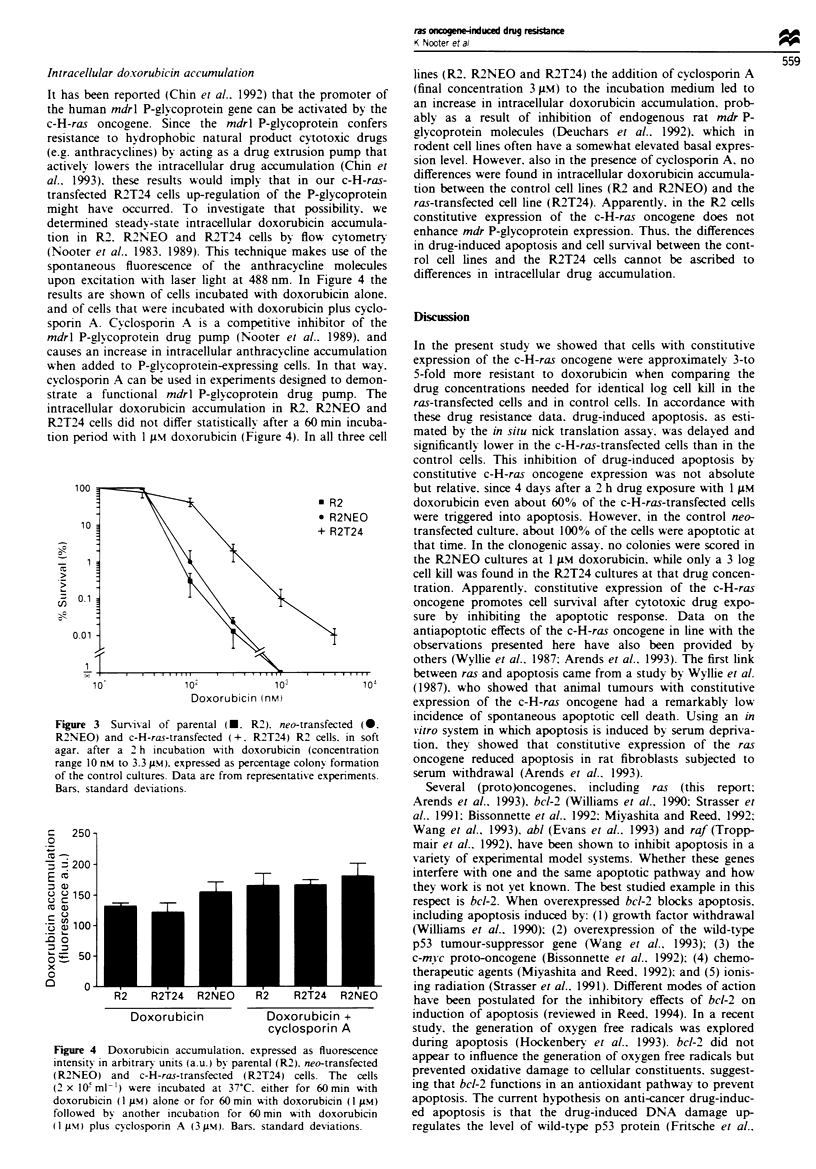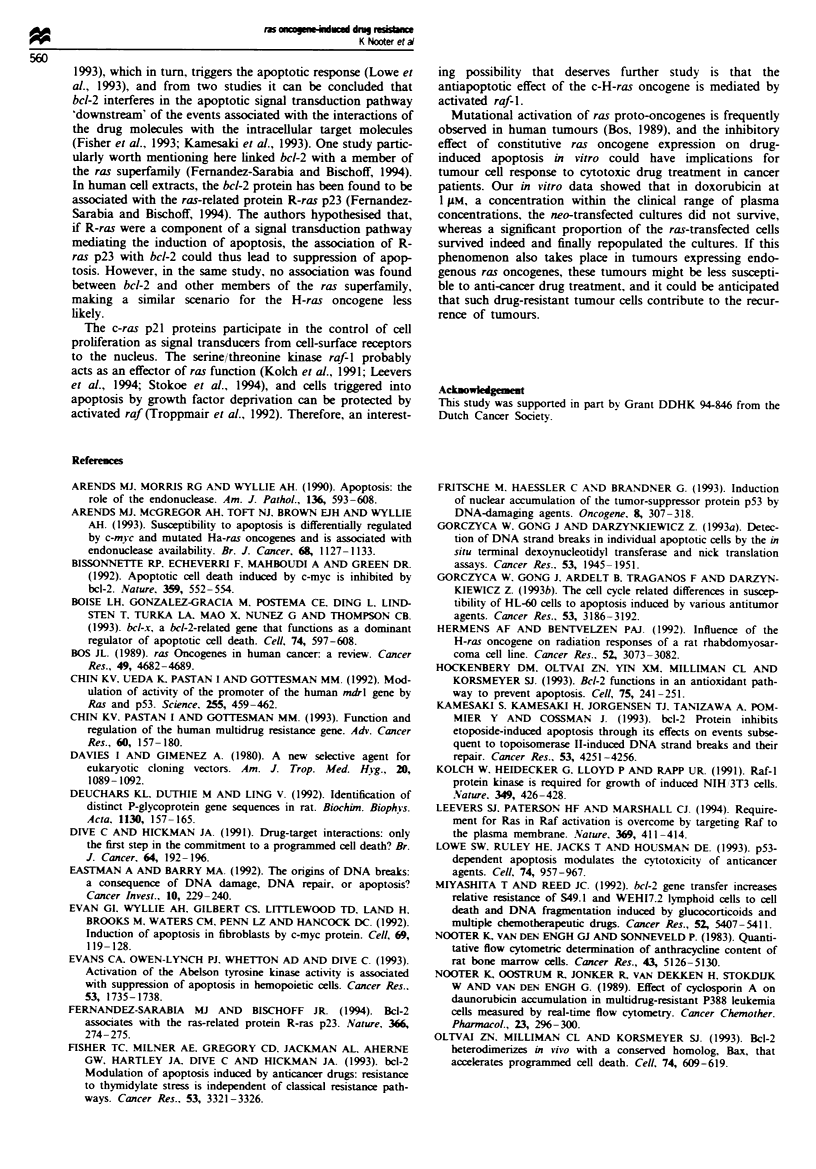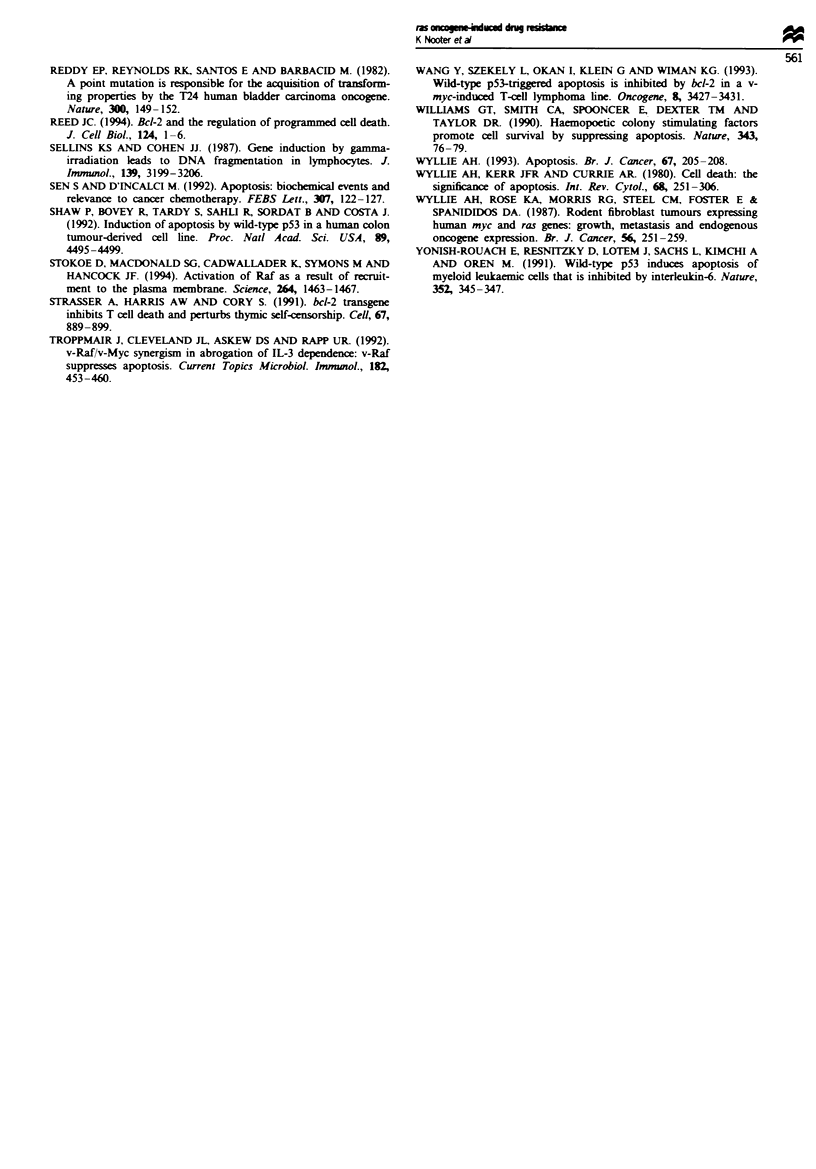# Constitutive expression of the c-H-ras oncogene inhibits doxorubicin-induced apoptosis and promotes cell survival in a rhabdomyosarcoma cell line.

**DOI:** 10.1038/bjc.1995.109

**Published:** 1995-03

**Authors:** K. Nooter, A. W. Boersma, R. G. Oostrum, H. Burger, A. G. Jochemsen, G. Stoter

**Affiliations:** Department of Medical Oncology, University Hospital Rotterdam, The Netherlands.

## Abstract

Drugs used in anti-cancer chemotherapy are thought to exert their cytotoxic action by induction of apoptosis. Genes have been identified which can mediate or modulate this drug-induced apoptosis, among which are c-myc, p53 and bcl-2. Since expression of oncogenic ras genes is a frequent observation in human cancer, we investigated the effects of the c-H-ras oncogene on anti-cancer drug-induced apoptosis. Apoptosis induced by a 2 h doxorubicin exposure was measured by in situ nick translation and flow cytometry in a rat cell line (R2T24) stably transfected with the c-H-ras oncogene and in a control cell line (R2NEO) transfected only with the antibiotic resistance gene neo. Both cell lines (R2T24 and R2NEO) had nearly identical growth characteristics, including cell doubling time, distribution over the cell cycle phases and plating efficiency in soft agar. Doxorubicin exposure of the R2NEO cells led to massive induction of apoptosis. In contrast, R2T24 cells, expressing the c-H-ras oncogene, showed significantly less apoptosis after doxorubicin incubation. Doxorubicin induced approximately 3- to 5-fold less cytotoxicity in the R2T24 cells than in the R2NEO cells, as determined by clonogenic assay in soft agar. No difference was observed in intracellular doxorubicin accumulation between the two cell lines, indicating that the classical, P-glycoprotein-mediated multidrug resistance phenotype is not involved in the observed differences in drug sensitivity. In conclusion, our data show that constitutive expression of the c-H-ras oncogene suppresses doxorubicin-induced apoptosis and promotes cell survival, suggesting that human tumours with ras oncogene expression might be less susceptible to doxorubicin treatment.


British Journal of Cancer (1995) 71, 556-561

© 1995 Stockton Press All rights reserved 0007-0920/95 $9.00

### Constitutive expression of the c-H-*ras* oncogene inhibits doxorubicin-induced apoptosis and promotes cell survival in a rhabdomyosarcoma cell line

K Nooter[1], AWM Boersma[1], RG Oostrum[1], H Burger[1], AG Jochemsen[2] and G Stoter[1]

[1]*Department of Medical Oncology, University Hospital Rotterdam and Rotterdam Cancer Institute (Daniel den Hoed Kliniek), Rotterdam;* [2]*Department of Medical Biochemistry, Leiden University, Leiden, The Netherlands.*

**Summary** Drugs used in anti-cancer chemotherapy are thought to exert their cytotoxic action by induction of apoptosis. Genes have been identified which can mediate or modulate this drug-induced apoptosis, among which are c-*myc*, p53 and *bcl*-2. Since expression of oncogenic *ras* genes is a frequent observation in human cancer, we investigated the effects of the c-H-*ras* oncogene on anti-cancer drug-induced apoptosis. Apoptosis induced by a 2 h doxorubicin exposure was measured by *in situ* nick translation and flow cytometry in a rat cell line (R2T24) stably transfected with the c-H-*ras* oncogene and in a control cell line (R2NEO) transfected only with the antibiotic resistance gene *neo*. Both cell lines (R2T24 and R2NEO) had nearly identical growth characteristics, including cell doubling time, distribution over the cell cycle phases and plating efficiency in soft agar. Doxorubicin exposure of the R2NEO cells led to massive induction of apoptosis. In contrast, R2T24 cells, expressing the c-H-*ras* oncogene, showed significantly less apoptosis after doxorubicin incubation. Doxorubicin induced approximately 3- to 5-fold less cytotoxicity in the R2T24 cells than in the R2NEO cells, as determined by clonogenic assay in soft agar. No difference was observed in intracellular doxorubicin accumulation between the two cell lines, indicating that the classical, P-glycoprotein-mediated multidrug resistance phenotype is not involved in the observed differences in drug sensitivity. In conclusion, our data show that constitutive expression of the c-H-*ras* oncogene suppresses doxorubicin-induced apoptosis and promotes cell survival, suggesting that human tumours with *ras* oncogene expression might be less susceptible to doxorubicin treatment.

**Keywords:** apoptosis; c-H-*ras* oncogene; drug resistance; doxorubicin

Chemotherapy failure due to cellular drug resistance is still a major problem in most cancer patients. A variety of drug resistance mechanisms have been characterised using *in vitro* cell lines made resistant to the different classes of anti-cancer agents. Qualitative and quantitative alterations in cellular target proteins, drug metabolism, repair mechanisms and drug efflux from the cell, among others, can cause drug resistance *in vitro*. However, a clear relationship between these cellular biochemical alterations and chemotherapy failure in patients could not be established for most drug resistance mechanisms identified so far. These resistance mechanisms have in common that they concern, directly or indirectly, the interaction of the drug molecule with its intracellular target molecules. A different approach for the elucidation of the mechanisms of cellular drug resistance is to study how cells are killed by cytotoxic drugs and to unravel the events that occur as a consequence of the drug-target interaction that finally leads to cell death.

It is now well appreciated that most anti-cancer drugs can exert their cytotoxic action by triggering a conserved, gene-activated programme for cell death, often referred to as apoptosis (Wyllie *et al*., 1980; Dive and Hickman, 1991; Eastman and Barry, 1992; Sen and D'Incalci, 1992; Wyllie, 1993). Apoptosis is the normal physiological method of cell death during, for example, embryogenesis and tissue homeostasis, and can also be induced by a large variety of external stimuli, such as viral infections and toxic insults. Therefore, it may well be that the susceptibility of a cancer cell to drug-induced apoptosis is an important determinant in the therapeutic response (Dive and Hickman, 1991). Recent evidence strongly suggests that modulation of the apoptotic cell response can lead to drug resistance. It has been shown that the *bcl*-2 gene can prevent or markedly reduce cell kill induced by anti-cancer drugs (Reed, 1994). This oncogene is a member of a superfamily of related genes, including *bax* (Oltvai *et al*., 1993) and *bcl-x* (Boise *et al*., 1993), which normally regulate apoptosis in mammalian cells and are thought to induce cytotoxic drug resistance by blocking a final common pathway to apoptotic cell death. Although the molecular mechanism of apoptosis is yet unknown, several other (onco)genes have been shown to mediate or modulate the apoptotic pathway, among which are the tumour-suppressor gene p53 and the proto-oncogene c-*myc*. In some cell systems, overexpression of these genes induces or facilitates apoptosis (Yonish-Rouach *et al*., 1991; Evan *et al*., 1992; Shaw *et al*., 1992). It is very likely that other (onco)genes involved in cell proliferation will also play a role in the process of apoptosis. Since oncogenic activation of the *ras* gene is frequently observed in human cancer (Bos, 1989), we investigated, in our effort to characterise drug resistance parameters in human tumours, the effects of the c-H-*ras* oncogene on chemotherapy-induced apoptosis.

### Materials and methods

### *Cell lines*

The rhabdomyosarcoma cell line R2 and the transfectants, R2T24 and R2NEO, have been described previously (Hermens and Bentvelzen, 1992), and were maintained in monolayer culture in Dulbecco's modified culture medium, supplemented with 10% fetal calf serum, 100 units $ml^{-1}$ penicillin, 100 $\mu g\ ml^{-1}$ streptomycin and 2 mM L-glutamine. The cells were cultured at 37°C in a humidified incubator with 8.5% carbon dioxide. The R2T24 cell line was co-transfected with the plasmid pT24 carrying the c-H-*ras* oncogene (Reddy *et al*., 1982) and the plasmid $pK_0$ carrying the *neo* gene (Davies and Gimenez, 1980). The R2NEO cell line was transfected with the *neo* gene only. Southern blot analysis with the 6.6 kb *Bam*HI fragment of the pT24 plasmid revealed that the R2T24 cell line contains six copies of the c-H-*ras* oncogene per cell (Hermens and Bentvelzen,

Correspondence: K Nooter, University Hospital Rotterdam, Department of Medical Oncology, Room 328, Dr Molewaterplein 40, 3015 GD, Rotterdam, The Netherlands
Received 5 July 1994; revised 14 October 1994; accepted 18 October 1994

1992). The R2T24 cells exhibit constitutive expression of the c-H-*ras* oncogene, as determined by dot-blot and Northern blot assay (Hermens and Bentvelzen, 1992).

For induction of apoptosis, the cells were seeded in 75 $cm^2$ flasks and 24 h later incubated for 2 h with doxorubicin at various concentrations, diluted in culture medium without serum. Thereafter, the cells were washed three times with phosphate-buffered saline (PBS) (pH 7.4) and further cultured in drug-free medium for either 24, 48, 72 or 96 h.

### *Quantification of apoptosis by* in situ *nick translation*

Several methods have been described for the detection of apoptotic cells, based on the various characteristics of the apoptotic response (Wyllie *et al.*, 1980). The landmark of apoptosis is endonucleolysis, a process whereby nuclear DNA is initially degraded at the nucleosomal linker regions (Arends *et al.*, 1990). Electrophoresis of such degraded DNA reveals a so-called ladder pattern of nucleosome-sized fragments of 180 kb, or a multiple of this. The DNA ladder technique is very often used for the detection of apoptotic cells. Unfortunately, this assay system is difficult to quantitate and cannot be used to evaluate apoptosis in individual cells. In the present study, we have used the quantitative *in situ* nick translation assay of Gorczyca *et al.* (1993*a*, *b*) for the detection of DNA breaks, in which the 3'-hydroxyl termini of DNA breaks are labelled with biotinylated dUTP by *Escherichia coli* DNA polymerase. The biotinylated dUTP molecules incorporated into the DNA can be quantiated by flow cytometry upon binding to fluoresceinated avidin. At different time points (24, 48, 72 and 96 h) after drug incubation, cells were fixed in 1% formaldehyde in PBS (pH 7.4) for 30 min on ice. After washing with PBS, the cells were resuspended in 70% ice-cold ethanol in PBS and stored at −20°C until further processing. For *in situ* nick translation the fixed cells were washed in PBS and resuspended in buffer containing 5 mM magnesium chloride, 10 mM β-mercaptoethanol, 50 mM Tris pH 7.8, 1 unit $ml^{-1}$ *E. coli* DNA polymerase, 0.2 nmol of biotin-11-dUTP and 0.2 nmol of unlabelled dATP, dCTP and dGTP. After incubation for 90 min at 15°C, the cells were washed with PBS supplemented with 0.1% Triton X-100, and resuspended in staining buffer consisting of 2.5 μg $ml^{-1}$ avidin–fluorescein isothiocyanate (FITC) in 4 × saline sodium citrate buffer (1 × SSC = 0.15 M sodium chloride, 0.015 M sodium citrate), 0.1% Triton X-100 and 5% (w/v) non-fat dry milk. Staining was performed for 30 min at 37°C. Thereafter, the cells were washed with PBS. DNA was counterstained with propidium iodide (1 μg $ml^{-1}$) for 30 min at 4°C in PBS. Flow cytometry was performed on a FACScan flow cytometer (Becton Dickinson) with excitation at 488 nm. The following parameters were measured: forward light scatter, perpendicular light scatter, FITC fluorescence (515–545 nm) and fluorescence of the DNA–propidium iodide complex (563–607 nm). Cell debris was excluded from analysis by appropriate forward light scatter threshold setting.

### *Cytotoxicity assay*

Doxorubicin-induced cytotoxicity was determined by colony formation in soft agar. Cells were incubated for 2 h with doxorubicin (concentration range 10 nM to 3.3 μM), washed twice and plated in triplicate at a density of $10^2$, $10^3$, $10^4$ and $10^5$ cells per 35 mm Petri dish in 1 ml of fresh medium containing 0.3% soft agar. Colonies were counted after 10 days of incubation at 37°C and 8.5% carbon dioxide.

### *Intracellular doxorubicin accumulation*

Intracellular doxorubicin accumulation was measured as described previously (Nooter *et al.*, 1983). Cellular anthracycline net uptake can be quantitated by flow cytometry by measuring the fluorescence of the anthracycline molecules upon excitation with laser light of 488 nm (Nooter *et al.*, 1983, 1989). The fluorescence which is emitted by the cells upon excitation by the laser light was registered on a photomultiplier of the FACScan flow cytometer. Data analysis was performed using histogram analysis of the LYSYS II software program (Becton Dickinson). The accumulation of doxorubicin was expressed in arbitrary units (a.u.) by calculating the mean fluorescence distribution of each cell sample. Cells (2 × $10^5$ $ml^{-1}$) in RPMI without phenol red buffered with 10 mM HEPES buffer (pH 7.4) were incubated at 37°C and 8.5% carbon dioxide either for 60 min with doxorubicin (1 μM) or for 60 min with doxorubicin (1 μM), followed by another incubation for 60 min after the addition of cyclosporin A (3 μM). The incubations were stopped by putting the cells on melting ice. After washing twice with ice-cold PBS (pH 7.4) the cells were stored at 4°C until flow cytometric analysis.

### Results

### *Growth characteristics of the cell lines*

Rat rhabdomyosarcoma R2 cells were stably transfected with plasmids containing the *ras* oncogene and/or the *neo* gene, resulting in the establishment of the cell lines R2T24 and R2NEO respectively. These cell lines have been described previously (Hermens and Bentvelzen, 1992), and their relevant growth characteristics will be summarised here. The R2T24 cell line contains six copies of the *ras* oncogene per cell, as estimated by Southern blot analysis. Dot-blot and Northern blot hybridisation showed abundant *ras* mRNA expression in R2T24 cells. The *in vitro* doubling time of the R2, R2T24 and R2NEO cell lines is 0.9, 1.0, and 1.0 days respectively. Cell cycle analysis of cells in logarithmic growth showed no differences between the R2, R2T24 and R2NEO cells in the distribution over the cell cycle phases. The mean proportions of cells in $G_1$, S and $G_2$/M were 50%, 31% and 19% for R2 cells, 54%, 30% and 16% for R2T24 cells and 56%, 28% and 16% for R2NEO cells.

### *Doxorubicin-induced apoptosis*

A 2 h incubation of exponentially growing R2 or R2NEO cells with doxorubicin (1 μM) resulted in an apoptotic response, as determined 24 h later by cell morphology, DNA degradation and *in situ* nick translation assay. The cells had a typical apoptotic feature with condensed chromatin and nuclear fragmentation. DNA degradation in nucleosome-sized fragments could be detected by qualitative gel electrophoresis (Sellins and Cohen, 1987) (data not shown). Prior to drug incubation, the R2 parental and the R2NEO cell lines only showed very low levels of spontaneous apoptosis, as determined by *in situ* nick translation assay (Figure 1a). However, after doxorubicin incubation apoptotic cells could be distinguished in R2 and R2NEO cultures on the basis of DNA content and biotin-dUTP labelling. In Figure 1b–1d, R2NEO cell cultures are shown at $t = 24$, $t = 48$ and $t = 72$ h after doxorubicin exposure. Extensive biotin-dUTP labelling was observed at $t = 24$ h (Figure 1b), which increased with time. At $t = 48$ h, two distinct apoptotic cell populations were present, representing about 70% of the total number of cells analysed. One cluster of apoptotic cells is only shifted on the vertical axis owing to incorporation of biotin-dUTP and has a normal DNA content. The other cluster of apoptotic cells has a reduced DNA content, probably as a result of loss of diffusible DNA of low molecular weight (200–1000 bp; mono- and short oligonucleosomes) (Gorczyca *et al.*, 1993*a*). At $t = 72$ h, the vast majority (about 90%) of cells were apoptotic, and formed one cluster with predominantly a subnormal DNA content (Figure 1d). In contrast, at $t =$ 24 h, R2T24 cells remained mostly viable after doxorubicin incubation and showed very little DNA degradation upon gel electrophoresis (data not shown). In the *in situ* nick translation assay a very small fraction (about 7%) of R2T24 cells was labelled above background (Figure 1e). Although, in the *ras*-transfected cultures the number of apoptotic cells also

**Figure 1** Labelling of DNA strand breaks with biotin-dUTP in control, *neo*-transfected (R2NEO) and c-H-*ras*-transfected (R2T24) R2 cells. The extent of DNA strand breaks is estimated by avidin–FITC fluorescence (ordinate) and cellular DNA content by DNA–propidium iodide fluorescence (abscissa), both expressed in arbitrary units (a.u.). The cells were treated with doxorubicin (1 μM) for 2 h and at the indicated time points thereafter labelled with biotin-dUTP by *in situ* nick translation and counterstained with propidium iodide. Control, untreated R2NEO cells. The position of cells in $G_1$, S, or $G_2 + M$ is indicated. AP, apoptotic cells. Data from representative experiments.

increased in time (up to about 40% at $t = 72$ h) (Figure 1f), there was a striking difference between the *ras*-transfected and the *neo*-transfected cells (compare Figure 1d and f).

Figure 2 shows the time course of drug-induced apoptosis in R2NEO and R2T24 cells at various doxorubicin concentrations. In the R2NEO cells the proportion of apoptotic cells increased with time at all drug concentrations tested, and the highest drug concentrations induced the highest percentages of apoptotic cells. At 1 μM doxorubicin – a concentration that gives more than 4 log cell kill in a clonogenic assay on the R2NEO cells (Figure 3) – 96 h after drug incubation practically all cells were apoptotic (Figure 2d). Compared with the *neo*-transfected cells, the apoptotic response in the *ras*-transfected cultures was clearly delayed and less extensive. Ninety-six hours after drug incubation (1 μM) a large population of cells with normal DNA content and only background biotin-dUTP labelling was still present in the R2T24 culture, and these surviving cells started to repopulate the culture flasks. In order to quantitate the differences in cell survival between R2NEO and R2T24 cells after doxorubicin incubation, we performed clonogenic assays.

### *Doxorubicin-induced cytotoxicity*

Doxorubicin-induced cytotoxicity was determined by colony formation in soft agar. Survival was expressed as percentage of colony formation in the control cultures, that is without drug incubation. In the control cultures the plating efficiencies varied between 80% and 90%, and no differences were found in this respect between the parental (R2) cells, the *neo*-transfected (R2NEO) cells and the c-H-*ras*-transfected (R2T24) cells. However, in the presence of doxorubicin the R2T24 cells were drug resistant by a factor of about 3–5 as compared with the R2 and R2NEO cells (Figure 3).

**Figure 2** Time course of doxorubicin-induced apoptosis in *neo*-transfected (R2NEO) (□) and c-H-*ras*-transfected (R2T24) (■) R2 cells at various drug concentrations. Apoptosis was assessed by flow cytometry as described previously (Gorczyca *et al.*, 1993*a*, *b*). The number of apoptotic cells is expressed as percentage of the total number of cells analysed (mean ± standard deviation of at least two independent experiments). Significant differences (Wilcoxon's signed-rank test, α = 0.05) in the percentage drug-induced apoptosis between R2NEO and R2T24 cells are indicated by an asterisk.

### Intracellular doxorubicin accumulation

It has been reported (Chin *et al*., 1992) that the promoter of the human *mdr*1 P-glycoprotein gene can be activated by the c-H-*ras* oncogene. Since the *mdr*1 P-glycoprotein confers resistance to hydrophobic natural product cytotoxic drugs (e.g. anthracyclines) by acting as a drug extrusion pump that actively lowers the intracellular drug accumulation (Chin *et al*., 1993), these results would imply that in our c-H-*ras*-transfected R2T24 cells up-regulation of the P-glycoprotein might have occurred. To investigate that possibility, we determined steady-state intracellular doxorubicin accumulation in R2, R2NEO and R2T24 cells by flow cytometry (Nooter *et al*., 1983, 1989). This technique makes use of the spontaneous fluorescence of the anthracycline molecules upon excitation with laser light at 488 nm. In Figure 4 the results are shown of cells incubated with doxorubicin alone, and of cells that were incubated with doxorubicin plus cyclosporin A. Cyclosporin A is a competitive inhibitor of the *mdr*1 P-glycoprotein drug pump (Nooter *et al*., 1989), and causes an increase in intracellular anthracycline accumulation when added to P-glycoprotein-expressing cells. In that way, cyclosporin A can be used in experiments designed to demonstrate a functional *mdr*1 P-glycoprotein drug pump. The intracellular doxorubicin accumulation in R2, R2NEO and R2T24 cells did not differ statistically after a 60 min incubation period with 1 μM doxorubicin (Figure 4). In all three cell lines (R2, R2NEO and R2T24) the addition of cyclosporin A (final concentration 3 μM) to the incubation medium led to an increase in intracellular doxorubicin accumulation, probably as a result of inhibition of endogenous rat *mdr* P-glycoprotein molecules (Deuchars *et al*., 1992), which in rodent cell lines often have a somewhat elevated basal expression level. However, also in the presence of cyclosporin A, no differences were found in intracellular doxorubicin accumulation between the control cell lines (R2 and R2NEO) and the *ras*-transfected cell line (R2T24). Apparently, in the R2 cells constitutive expression of the c-H-*ras* oncogene does not enhance *mdr* P-glycoprotein expression. Thus, the differences in drug-induced apoptosis and cell survival between the control cell lines and the R2T24 cells cannot be ascribed to differences in intracellular drug accumulation.

### Discussion

In the present study we showed that cells with constitutive expression of the c-H-*ras* oncogene were approximately 3-to 5-fold more resistant to doxorubicin when comparing the drug concentrations needed for identical log cell kill in the *ras*-transfected cells and in control cells. In accordance with these drug resistance data, drug-induced apoptosis, as estimated by the *in situ* nick translation assay, was delayed and significantly lower in the c-H-*ras*-transfected cells than in the control cells. This inhibition of drug-induced apoptosis by constitutive c-H-*ras* oncogene expression was not absolute but relative, since 4 days after a 2 h drug exposure with 1 μM doxorubicin even about 60% of the c-H-*ras*-transfected cells were triggered into apoptosis. However, in the control *neo*-transfected culture, about 100% of the cells were apoptotic at that time. In the clonogenic assay, no colonies were scored in the R2NEO cultures at 1 μM doxorubicin, while only a 3 log cell kill was found in the R2T24 cultures at that drug concentration. Apparently, constitutive expression of the c-H-*ras* oncogene promotes cell survival after cytotoxic drug exposure by inhibiting the apoptotic response. Data on the antiapoptotic effects of the c-H-*ras* oncogene in line with the observations presented here have also been provided by others (Wyllie *et al*., 1987; Arends *et al*., 1993). The first link between *ras* and apoptosis came from a study by Wyllie *et al*. (1987), who showed that animal tumours with constitutive expression of the c-H-*ras* oncogene had a remarkably low incidence of spontaneous apoptotic cell death. Using an *in vitro* system in which apoptosis is induced by serum deprivation, they showed that constitutive expression of the *ras* oncogene reduced apoptosis in rat fibroblasts subjected to serum withdrawal (Arends *et al*., 1993).

Several (proto)oncogenes, including *ras* (this report; Arends *et al*., 1993), *bcl*-2 (Williams *et al*., 1990; Strasser *et al*., 1991; Bissonnette *et al*., 1992; Miyashita and Reed, 1992; Wang *et al*., 1993), *abl* (Evans *et al*., 1993) and *raf* (Troppmair *et al*., 1992), have been shown to inhibit apoptosis in a variety of experimental model systems. Whether these genes interfere with one and the same apoptotic pathway and how they work is not yet known. The best studied example in this respect is *bcl*-2. When overexpressed *bcl*-2 blocks apoptosis, including apoptosis induced by: (1) growth factor withdrawal (Williams *et al*., 1990); (2) overexpression of the wild-type p53 tumour-suppressor gene (Wang *et al*., 1993); (3) the c-*myc* proto-oncogene (Bissonnette *et al*., 1992); (4) chemotherapeutic agents (Miyashita and Reed, 1992); and (5) ionising radiation (Strasser *et al*., 1991). Different modes of action have been postulated for the inhibitory effects of *bcl*-2 on induction of apoptosis (reviewed in Reed, 1994). In a recent study, the generation of oxygen free radicals was explored during apoptosis (Hockenbery *et al*., 1993). *bcl*-2 did not appear to influence the generation of oxygen free radicals but prevented oxidative damage to cellular constituents, suggesting that *bcl*-2 functions in an antioxidant pathway to prevent apoptosis. The current hypothesis on anti-cancer drug-induced apoptosis is that the drug-induced DNA damage upregulates the level of wild-type p53 protein (Fritsche *et al*.,

1993), which in turn, triggers the apoptotic response (Lowe *et al.*, 1993), and from two studies it can be concluded that *bcl*-2 interferes in the apoptotic signal transduction pathway 'downstream' of the events associated with the interactions of the drug molecules with the intracellular target molecules (Fisher *et al.*, 1993; Kamesaki *et al.*, 1993). One study particularly worth mentioning here linked *bcl*-2 with a member of the *ras* superfamily (Fernandez-Sarabia and Bischoff, 1994). In human cell extracts, the *bcl*-2 protein has been found to be associated with the *ras*-related protein R-*ras* p23 (Fernandez-Sarabia and Bischoff, 1994). The authors hypothesised that, if R-*ras* were a component of a signal transduction pathway mediating the induction of apoptosis, the association of R-*ras* p23 with *bcl*-2 could thus lead to suppression of apoptosis. However, in the same study, no association was found between *bcl*-2 and other members of the *ras* superfamily, making a similar scenario for the H-*ras* oncogene less likely.

The c-*ras* p21 proteins participate in the control of cell proliferation as signal transducers from cell-surface receptors to the nucleus. The serine/threonine kinase *raf*-1 probably acts as an effector of *ras* function (Kolch *et al.*, 1991; Leevers *et al.*, 1994; Stokoe *et al.*, 1994), and cells triggered into apoptosis by growth factor deprivation can be protected by activated *raf* (Troppmair *et al.*, 1992). Therefore, an interesting possibility that deserves further study is that the antiapoptotic effect of the c-H-*ras* oncogene is mediated by activated *raf*-1.

Mutational activation of *ras* proto-oncogenes is frequently observed in human tumours (Bos, 1989), and the inhibitory effect of constitutive *ras* oncogene expression on drug-induced apoptosis *in vitro* could have implications for tumour cell response to cytotoxic drug treatment in cancer patients. Our *in vitro* data showed that in doxorubicin at 1 μM, a concentration within the clinical range of plasma concentrations, the *neo*-transfected cultures did not survive, whereas a significant proportion of the *ras*-transfected cells survived indeed and finally repopulated the cultures. If this phenomenon also takes place in tumours expressing endogenous *ras* oncogenes, these tumours might be less susceptible to anti-cancer drug treatment, and it could be anticipated that such drug-resistant tumour cells contribute to the recurrence of tumours.

**Acknowledgement**
This study was supported in part by Grant DDHK 94-846 from the Dutch Cancer Society.

**References**

ARENDS MJ, MORRIS RG AND WYLLIE AH. (1990). Apoptosis: the role of the endonuclease. *Am. J. Pathol.*, **136**, 593-608.
ARENDS MJ, MCGREGOR AH, TOFT NJ, BROWN EJH AND WYLLIE AH. (1993). Susceptibility to apoptosis is differentially regulated by c-*myc* and mutated Ha-*ras* oncogenes and is associated with endonuclease availability. *Br. J. Cancer*, **68**, 1127-1133.
BISSONNETTE RP, ECHEVERRI F, MAHBOUDI A AND GREEN DR. (1992). Apoptotic cell death induced by c-myc is inhibited by bcl-2. *Nature*, **359**, 552-554.
BOISE LH, GONZALEZ-GRACIA M, POSTEMA CE, DING L, LINDSTEN T, TURKA LA, MAO X, NUNEZ G AND THOMPSON CB. (1993). *bcl-x*, a *bcl*-2-related gene that functions as a dominant regulator of apoptotic cell death. *Cell*, **74**, 597-608.
BOS JL. (1989). *ras* Oncogenes in human cancer: a review. *Cancer Res.*, **49**, 4682-4689.
CHIN KV, UEDA K, PASTAN I AND GOTTESMAN MM. (1992). Modulation of activity of the promoter of the human *mdr*1 gene by *Ras* and p53. *Science*, **255**, 459-462.
CHIN KV, PASTAN I AND GOTTESMAN MM. (1993). Function and regulation of the human multidrug resistance gene. *Adv. Cancer Res.*, **60**, 157-180.
DAVIES I AND GIMENEZ A. (1980). A new selective agent for eukaryotic cloning vectors. *Am. J. Trop. Med. Hyg.*, **20**, 1089-1092.
DEUCHARS KL, DUTHIE M AND LING V. (1992). Identification of distinct P-glycoprotein gene sequences in rat. *Biochim. Biophys. Acta*, **1130**, 157-165.
DIVE C AND HICKMAN JA. (1991). Drug-target interactions: only the first step in the commitment to a programmed cell death? *Br. J. Cancer*, **64**, 192-196.
EASTMAN A AND BARRY MA. (1992). The origins of DNA breaks: a consequence of DNA damage, DNA repair, or apoptosis? *Cancer Invest.*, **10**, 229-240.
EVAN GI, WYLLIE AH, GILBERT CS, LITTLEWOOD TD, LAND H, BROOKS M, WATERS CM, PENN LZ AND HANCOCK DC. (1992). Induction of apoptosis in fibroblasts by c-myc protein. *Cell*, **69**, 119-128.
EVANS CA, OWEN-LYNCH PJ, WHETTON AD AND DIVE C. (1993). Activation of the Abelson tyrosine kinase activity is associated with suppression of apoptosis in hemopoietic cells. *Cancer Res.*, **53**, 1735-1738.
FERNANDEZ-SARABIA MJ AND BISCHOFF JR. (1994). Bcl-2 associates with the ras-related protein R-ras p23. *Nature*, **366**, 274-275.
FISHER TC, MILNER AE, GREGORY CD, JACKMAN AL, AHERNE GW, HARTLEY JA, DIVE C AND HICKMAN JA. (1993). bcl-2 Modulation of apoptosis induced by anticancer drugs: resistance to thymidylate stress is independent of classical resistance pathways. *Cancer Res.*, **53**, 3321-3326.
FRITSCHE M, HAESSLER C AND BRANDNER G. (1993). Induction of nuclear accumulation of the tumor-suppressor protein p53 by DNA-damaging agents. *Oncogene*, **8**, 307-318.
GORCZYCA W, GONG J AND DARZYNKIEWICZ Z. (1993*a*). Detection of DNA strand breaks in individual apoptotic cells by the *in situ* terminal dexoynucleotidyl transferase and nick translation assays. *Cancer Res.*, **53**, 1945-1951.
GORCZYCA W, GONG J, ARDELT B, TRAGANOS F AND DARZYNKIEWICZ Z. (1993*b*). The cell cycle related differences in susceptibility of HL-60 cells to apoptosis induced by various antitumor agents. *Cancer Res.*, **53**, 3186-3192.
HERMENS AF AND BENTVELZEN PAJ. (1992). Influence of the H-*ras* oncogene on radiation responses of a rat rhabdomyosarcoma cell line. *Cancer Res.*, **52**, 3073-3082.
HOCKENBERY DM, OLTVAI ZN, YIN XM, MILLIMAN CL AND KORSMEYER SJ. (1993). *Bcl*-2 functions in an antioxidant pathway to prevent apoptosis. *Cell*, **75**, 241-251.
KAMESAKI S, KAMESAKI H, JORGENSEN TJ, TANIZAWA A, POMMIER Y AND COSSMAN J. (1993). bcl-2 Protein inhibits etoposide-induced apoptosis through its effects on events subsequent to topoisomerase II-induced DNA strand breaks and their repair. *Cancer Res.*, **53**, 4251-4256.
KOLCH W, HEIDECKER G, LLOYD P AND RAPP UR. (1991). Raf-1 protein kinase is required for growth of induced NIH/3T3 cells. *Nature*, **349**, 426-428.
LEEVERS SJ, PATERSON HF AND MARSHALL CJ. (1994). Requirement for Ras in Raf activation is overcome by targeting Raf to the plasma membrane. *Nature*, **369**, 411-414.
LOWE SW, RULEY HE, JACKS T AND HOUSMAN DE. (1993). p53-dependent apoptosis modulates the cytotoxicity of anticancer agents. *Cell*, **74**, 957-967.
MIYASHITA T AND REED JC. (1992). *bcl*-2 gene transfer increases relative resistance of S49.1 and WEHI7.2 lymphoid cells to cell death and DNA fragmentation induced by glucocorticoids and multiple chemotherapeutic drugs. *Cancer Res.*, **52**, 5407-5411.
NOOTER K, VAN DEN ENGH GJ AND SONNEVELD P. (1983). Quantitative flow cytometric determination of anthracycline content of rat bone marrow cells. *Cancer Res.*, **43**, 5126-5130.
NOOTER K, OOSTRUM R, JONKER R, VAN DEKKEN H, STOKDIJK W AND VAN DEN ENGH G. (1989). Effect of cyclosporin A on daunorubicin accumulation in multidrug-resistant P388 leukemia cells measured by real-time flow cytometry. *Cancer Chemother. Pharmacol.*, **23**, 296-300.
OLTVAI ZN, MILLIMAN CL AND KORSMEYER SJ. (1993). Bcl-2 heterodimerizes *in vivo* with a conserved homolog, Bax, that accelerates programmed cell death. *Cell*, **74**, 609-619.

REDDY EP, REYNOLDS RK, SANTOS E AND BARBACID M. (1982). A point mutation is responsible for the acquisition of transforming properties by the T24 human bladder carcinoma oncogene. *Nature*, **300**, 149-152.

REED JC. (1994). *Bcl*-2 and the regulation of programmed cell death. *J. Cell Biol.*, **124**, 1-6.

SELLINS KS AND COHEN JJ. (1987). Gene induction by gamma-irradiation leads to DNA fragmentation in lymphocytes. *J. Immunol.*, **139**, 3199-3206.

SEN S AND D'INCALCI M. (1992). Apoptosis: biochemical events and relevance to cancer chemotherapy. *FEBS Lett.*, **307**, 122-127.

SHAW P, BOVEY R, TARDY S, SAHLI R, SORDAT B AND COSTA J. (1992). Induction of apoptosis by wild-type p53 in a human colon tumour-derived cell line. *Proc. Natl Acad. Sci. USA*, **89**, 4495-4499.

STOKOE D, MACDONALD SG, CADWALLADER K, SYMONS M AND HANCOCK JF. (1994). Activation of Raf as a result of recruitment to the plasma membrane. *Science*, **264**, 1463-1467.

STRASSER A, HARRIS AW AND CORY S. (1991). *bcl*-2 transgene inhibits T cell death and perturbs thymic self-censorship. *Cell*, **67**, 889-899.

TROPPMAIR J, CLEVELAND JL, ASKEW DS AND RAPP UR. (1992). v-Raf/v-Myc synergism in abrogation of IL-3 dependence: v-Raf suppresses apoptosis. *Current Topics Microbiol. Immunol.*, **182**, 453-460.

WANG Y, SZEKELY L, OKAN I, KLEIN G AND WIMAN KG. (1993). Wild-type p53-triggered apoptosis is inhibited by *bcl*-2 in a v-*myc*-induced T-cell lymphoma line. *Oncogene*, **8**, 3427-3431.

WILLIAMS GT, SMITH CA, SPOONCER E, DEXTER TM AND TAYLOR DR. (1990). Haemopoetic colony stimulating factors promote cell survival by suppressing apoptosis. *Nature*, **343**, 76-79.

WYLLIE AH. (1993). Apoptosis. *Br. J. Cancer*, **67**, 205-208.

WYLLIE AH, KERR JFR AND CURRIE AR. (1980). Cell death: the significance of apoptosis. *Int. Rev. Cytol.*, **68**, 251-306.

WYLLIE AH, ROSE KA, MORRIS RG, STEEL CM, FOSTER E & SPANIDIDOS DA. (1987). Rodent fibroblast tumours expressing human *myc* and *ras* genes: growth, metastasis and endogenous oncogene expression. *Br. J. Cancer*, **56**, 251-259.

YONISH-ROUACH E, RESNITZKY D, LOTEM J, SACHS L, KIMCHI A AND OREN M. (1991). Wild-type p53 induces apoptosis of myeloid leukaemic cells that is inhibited by interleukin-6. *Nature*, **352**, 345-347.